# Consolidation of pathology services in England: have savings been achieved?

**DOI:** 10.1186/s12913-018-3683-8

**Published:** 2018-11-15

**Authors:** Giovanni Satta, John Edmonstone

**Affiliations:** 1 Imperial College Healthcare NHS Trust, London, UK; 2 School of Public Policy & Professional Practice, University of Keele, Ripon, UK

**Keywords:** Consolidation of pathology services, Savings, England

## Abstract

**Background:**

During the last decade, pathology services in England have undergone profound changes with an extensive consolidation of laboratories. This has been driven by some national reviews forecasting a national reduction of costs by £250–£500 million ($315–$630 million) a year as a result.

The main aim of this paper is to describe the financial impact of such consolidation, with a specific focus on the forecasted savings. A secondary aim is to describe the development of private sector involvement in laboratory services in a traditionally publicly funded healthcare system and the development of pathology staff size.

**Methods:**

In the English scenario, the majority of hospitals and laboratories are publicly funded and a survey was sent as Freedom of Information request to all directors of pathology. A descriptive comparison of savings among consolidated and non-consolidated pathology services was made by using the pathology budgets in two different periods (2015 versus 2010), adjusted by inflation and increased activity.

**Results:**

The *hub-and-spoke* model has been implemented as part of the consolidation process of pathology services in England. Consolidated pathology networks have achieved higher savings compared to non-consolidated single laboratories. There has been an increased role of private providers and savings were achieved with negligible personnel redundancies.

**Conclusions:**

Consolidated units have on average achieved larger cost savings than non-consolidated units but further analysis with stronger research design is required to independently evaluate the impact of pathology consolidation on both savings and quality.

## Background

Pathology services are an essential component of any healthcare system as they provide all laboratory tests for any patient in and out the hospital settings. They are represented by the main specialities of clinical biochemistry, haematology, blood transfusion, microbiology and histopathology.

Different countries around the globe have undergone consolidation of their pathology services in an attempt to reduce costs, meet increasing demand and achieve efficiency savings whilst modernizing laboratory infrastructure with new technologies. However, there is a paucity of published data regarding the effects of merging pathology laboratories, in particular the financial benefits following a wider national policy of consolidation. Most of the current evidence in healthcare is based on the benefits of merging entire hospitals. Classical examples include the Mayo and Cleveland clinics in the United States (US) [1, 2]: they have grown through mergers to become some of the most highly regarded hospital systems in the world. More recent evidence from a large sample of US hospital mergers between 2000 and 2010 found evidence of economically and statistically significant cost reductions at acquired hospitals [3]. University College London Hospitals (UCLH), created in 2004 from the merger of six different hospitals, is the best example in the United Kingdom (UK) [4]. UCLH has achieved a strong market share in several key specialties. It scores highly in national rankings of care quality and patient satisfaction and has sustained robust financial performance. However, these examples may not directly translate to pathology services as previous reports [5, 6] suggest that the cost savings achieved are typically in back-office and from the reduction of management functions rather than clinical costs, whilst savings in the pathology sector may results from the economy of scale and higher purchase discounts. These examples may also represent isolated success stories as an earlier analysis of more than 700 US hospital mergers by McKinsey and the London School of Economics [7] found that the cost savings achieved were substantially less than the original anticipated benefits. Similar findings from the analysis of more than 100 UK hospital mergers [8] have found that in many sites clinical productivity remained unchanged and the financial performance deteriorated, with none of them enhancing the quality of care. This is also confirmed by a report from Norway [9] where they discovered that only one hospital out of 17 actually increased its cost efficiency.

### Consolidation of pathology services in US

There are some successful examples of laboratory consolidation from the US [10]. The decline of the reimbursement rate since 1980 and cuts under the Clinical Laboratory Fee Schedule (CLFS) have had a profound impact on US laboratories, making pathology services less profitable and forcing many providers to merge. Between 2010 and 2013, CLFS reimbursement rates were effectively cut by nearly 12% [11]. A recent paper on the consolidation of clinical microbiology laboratories [12] highlights how, in the state of North Carolina (with a total population of around 10 million), five hospital systems dominate the health care landscape, with catchment areas exceeding hundreds of miles. The main core microbiology laboratory covers up to 40 different hospitals and its consolidation has achieved decreased turn-around-times (TATs) and reductions of costs, as a consequence of discount when purchasing in high volume. Conversely, earlier evidence suggested that TATs can be a major issue in the consolidated model [13], so the impact of consolidating laboratories on patient care still needs to be evaluated.

### Consolidation of pathology services in Australia

An interesting report from the Grattan Institute, a public policy think tank, [14], has highlighted impressive efficiency savings with the consolidation of Australian pathology services, and with two private providers dominating the market. However, this privatization has not been exempted from criticism, with accusation of not re-investing such savings into the public healthcare system and suggesting the government to introduce price competition into the market.

### Consolidation of pathology services in Europe

The best example of consolidation of pathology services in Europe is represented by Germany. Around 15 years ago, Germany had approximately 800 pathology laboratories, most of which were publicly funded [15]. Due to running costs of the services, since 1991 radical reforms of the system have seen bed capacity fall by almost a fifth and the number of hospitals by 9% [16] (including a 35% reduction in the number of pathology laboratories) and the German system has achieved a progressive reduction of tariffs equivalent to circa 40% of English reference costs for similar tests. This was also possible due to the fact that the tariff for pathology services is actually fixed, so the various providers can only compete on quality of service and clinical advice, rather than costs. The pathology landscape in Germany is now very different, with the majority of District General Hospitals (DGH) only equipped with a core laboratory for urgent testing (i.e. result needed in less than 2 h) and scientists cross trained in all pathology specialties. The majority of these core laboratories are part of wider pathology networks (largely privatized) and with the predominance of five private providers covering up to 60% of the German pathology market.

### Consolidation of pathology services in England

The National Health Service (NHS) is the publicly funded national healthcare system in England and the biggest of the four National Health Services of the UK, catering to a population of 54.3 million and employing around 1.2 million people. It is the largest single-payer healthcare system in the world with the majority of services completely free at the point of use and with an overall budget of £101.3 billion (around $128 billion) (NHS England data for 2015/16) [17]. Please note that similar healthcare systems are available in Scotland, Wales and Northern Ireland. During the last decade, pathology services in England have undergone profound changes. In particular, the *Carter review* [18] was published in 2006 and it highlighted the importance of pathology services (with an estimated 70–80% of all healthcare decisions affecting diagnosis or treatment being influenced by laboratory medicine results) but also its high cost (£2.5–$3.15 billion per annum). It also showed a wide variation of prices across the country for the same identical tests (but performed in different laboratories) with uncontrolled budgeting and rising costs. The solution proposed was a large-scale service consolidation of different laboratories with possible savings of £4–5 million ($5–6.3 million) by one network only (in England, there were around 150 hospitals offering pathology services). A second review, following various pilot projects, was published in 2010 [19] and it confirmed the strong case for consolidation of pathology services to improve quality, patient safety and efficiency, with a *hub-and-spoke* model (a central hub, where the majority of non-urgent tests can be performed, and district spokes, to process urgent specimens). Of note, in the foreword of this document, a total national reduction of costs by £250–£500 million ($315–£630 million) a year was forecasted as a result of consolidation.

Hence, the main aim of this paper is to compare the development of costs in consolidated and non-consolidated units in England, in particular assessing the achievement of the forecasted savings. A secondary aim is the description of private sector involvement in a traditionally publicly funded healthcare system and the impact on pathology staff in terms of personnel size and redundancies.

## Methods

A survey was sent as a Freedom of Information (FOI) request to all directors of pathology laboratories in English NHS hospitals. The FOI (based on the Freedom of Information Act 2000) allows any citizen to access information held by public authorities (in the English scenario, the majority of hospitals are publicly funded). A FOI request was chosen as the best method to obtain the necessary information. This was a reasoned decision with two main considerations in mind: it would have been very difficult to find the contact details of all directors of pathology in England and the response rate would have been affected and much lower.

### Selection of NHS hospitals and data collection

The survey focused on pathology services in two separate periods, 2010 and 2015, collecting information on the presence of laboratories on site, the total pathology budget, consolidation (existing or planned), the role of private providers (if any), and the number of staff (including redundancies).

The original list included all acute NHS hospitals in England and it was taken from the NHS Choice website [20]. Mental health hospitals and community providers were excluded as they do not have their own pathology services. Historically, the majority of hospitals used to have their own laboratory and pathology services on site. To avoid missing important information and taking into consideration all the recent changes and mergers in the NHS scenario, the FOI included a specific question on how many hospitals the NHS organization (also called NHS Trust/Foundation Trust) were currently managing and if those hospitals used to have separate pathology services in the year 2010. The original list included 160 names but it has been reduced to 153 as two mental health hospitals were excluded and five other NHS Trusts did not exist anymore (acquired or merged with another). Please note that NHS Trusts/Foundation Trusts may include different hospitals as part of their network.

### Analysis of data

A descriptive calculation of savings among consolidated and non-consolidated pathology services was made by comparing two different periods (2015 versus 2010). However, a direct comparison of the two budgets would have not been reasonable as the inflation and increased activity should also be taken into consideration. The 2010 budget was then adjusted using the inflation calculator provided by the Bank of England (inflation averaged 3.0% a year) [21]. Based on The King’s Fund data, the NHS has constantly increased its activity in the last decade [22]. A conservative assumption was made that during those 5 years there was an increased laboratory activity of 10% and such percentage of the 2015 budget was added when calculating savings. A statistical significance using the *t-test* was performed to compare the average savings. Additionally, a relative comparison based on the total savings divided by the size of the laboratories (number of personnel) has also been performed to augment the analysis and validate the statistical significance.

## Results

### Response rate and missing data

Complete information was obtained for 135 out of 153 (88%) organizations. Incomplete information was obtained for 18 organizations as fifteen NHS Trusts/Foundation Trusts were unable to provide the information for both periods and three organizations did not respond to the FOI request. However, they were still included in the initial epidemiological analysis but not in the budget comparison.

### Number of centralized laboratories in 2010 and 2015 (*n* = 153)

**Fig. 1:**
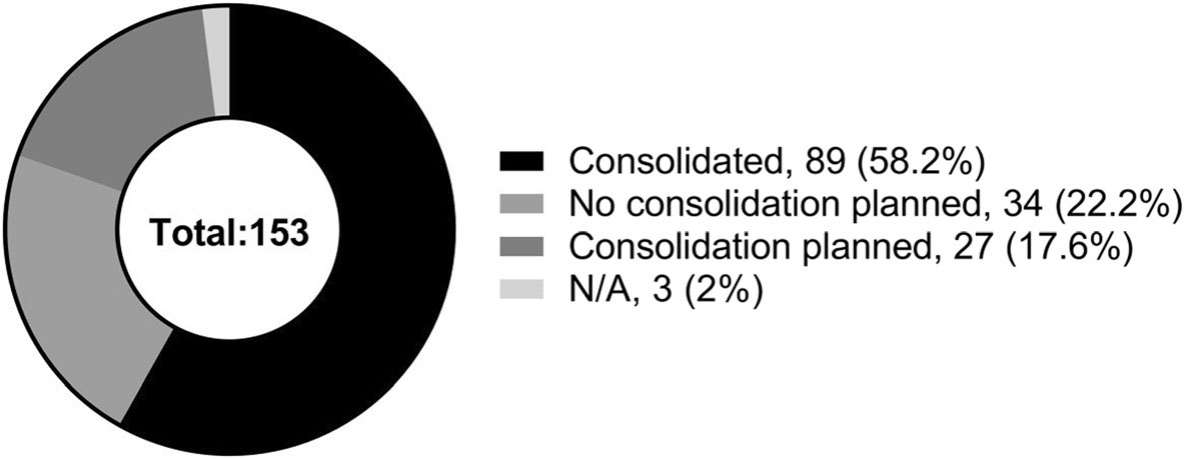
Centralization of laboratories in England. The majority of laboratory in England are already consolidated (*n* = 89 out of a total of 153). Around half of the remaining non-consolidated laboratories (*n* = 27) are planning a consolidation in the near future

At the time of the FOI request (end of 2016), the majority of Trusts in England (*n* = 89–58.2% of the total) had already gone through a process of consolidating their pathology services. Many of these Trusts now cover different hospitals (i.e. Imperial Healthcare includes St Mary’s, Hammersmith and Charing Cross Hospitals, all of which used to have their own separate pathology laboratories) and many of them share their pathology services with neighbouring partners (i.e., North West London Pathology covers Imperial Healthcare, Chelsea and Westminster and the Hillingdon hospitals). However, there is still a significant number of Trusts (*n* = 61–39.8% of the total) that have not yet had any consolidation of their pathology services. Around half of them (*n* = 27–17.6% of the total) are planning a consolidation of their pathology services in the near future (Fig. 1).

### Comparison of consolidated versus non-consolidated laboratories (*n* = 135)

Complete information was obtained for 135 NHS Trusts (but covered by 113 laboratories), 78 with consolidated pathology services and 57 with independent single laboratories. Those 78 organizations had consolidated their laboratory services into 56 pathology hubs, with an average size of 278 employees per hub (total number: 15,550). On the other hand, the 57 single laboratories have an average size of 155 employees per laboratory (total number: 8835). The budget information is shown in Table 1:

- There would be no cost savings at all in both consolidated and non-consolidated laboratories by simply comparing the total budgets 2010 versus 2015. The total national budget for 2015 is higher than the 2010 budget.
- However, after adjusting the 2010 budget for inflation, it is possible to calculate total national savings (if any) by detracting the 2015 budget. The 56 pathology hubs (consolidated laboratories) have saved £76,717,848. On the other hand, the non-consolidated laboratories have not made any savings and lost £825,471.
- When including the increased activity (10% of 2015 budget), the combined consolidated laboratories have saved £188,191,500 compared to £73,065,515 of non-consolidated laboratories.
- When calculating the average saving per laboratory, consolidated hubs have saved £3,360,562 each, whilst non-consolidated laboratories have saved £1,281,851 each. This difference is statistically significant (*p* = 0.0003).
- When calculating the relative saving per member of staff, consolidated hubs have saved £12,102 per employee versus £8270 per employee in a non-consolidated laboratory. This difference is again statistically significant (*p* = 0.0001).

**Table 1:** Comparison of total budgets: 2010, with and without inflation, versus 2015

Consolidated laboratories (*n* = 56 pathology hubs)				Non-consolidated laboratories (*n* = 57 single laboratories)			
Total budget 2010	Total budget 2015	Total budget 2010 with inflation	Difference (savings or deficit)	Total budget 2010	Total budget 2015	Total budget 2010 with inflation	Difference (savings or deficit)
£1,030,140,845	£1,114,736,579	£1,191,454,427	£76,717,848 (savings)	£631,153,715	£738,909,949	£738,084,478	-£825,471 (deficit)
Value of 10% increased activity: £111,473,652				Value of 10% increased activity: £73,890,986			
Total savings including increased activity: £188,191,500				Total savings including increased activity: £73,065,515			
Average savings per pathology hub				Average savings per single laboratory			
£3,360,562 (SD: £3,672,787)				£1,281,851 (SD: £2,074,070)			
*p* = 0.0003							
Average size of pathology hub (number of staff): 278				Average size of single laboratory (number of staff): 155			
Relative saving per member of staff:				Relative saving per member of staff:			
£12,102 (SD: £236)				£8270 (SD: £235)			
*p* = 0.0001							

Savings (total, average and per employee) and size of consolidated laboratories versus non-consolidated are also shown. All sums are in British sterling (£). SD = standard deviation. Not all laboratories have achieved savings and this explains the wide standard deviation observed in both groups

**Fig. 2:**
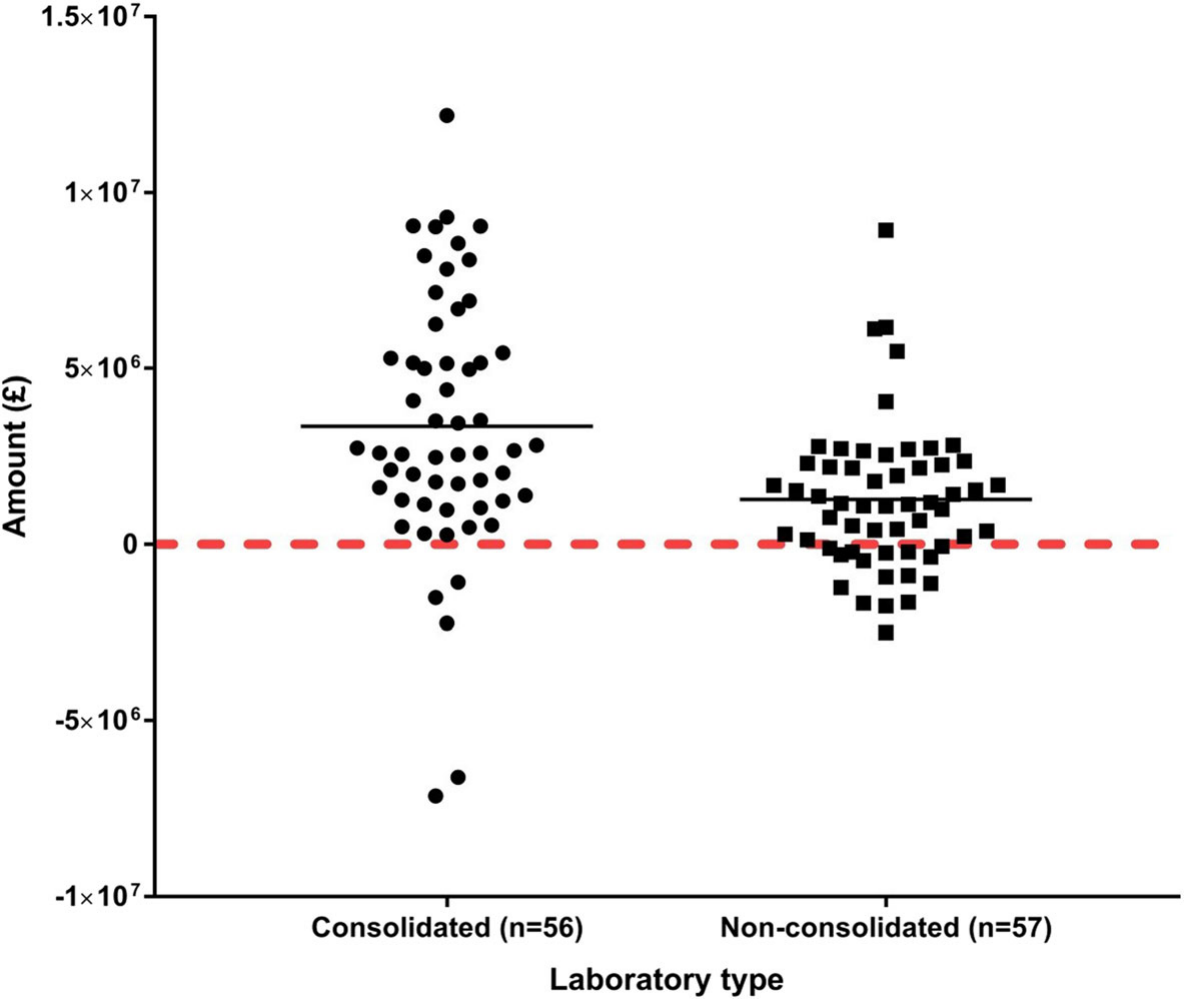
Consolidated versus non-consolidated laboratories. Graphical comparison of savings and deficits in consolidated (*n* = 56) versus non-consolidated (*n* = 57) laboratories. Each dot represents a laboratory (hub or single). Not all laboratories have achieved savings and this explains the negative values above (values under the red line). The two black lines indicate the mean or average savings per laboratory

It is important to notice that not all laboratories in both groups (consolidated and non-consolidated) have made savings. Whilst some laboratories have achieved high savings in their budget, others have experienced significant losses. In particular, five consolidated and fifteen non-consolidated laboratories have recorded a deficit in their budget. A graphical comparison of savings and deficits is shown in Fig. 2.

### Number and share of private providers

**Fig. 3:**
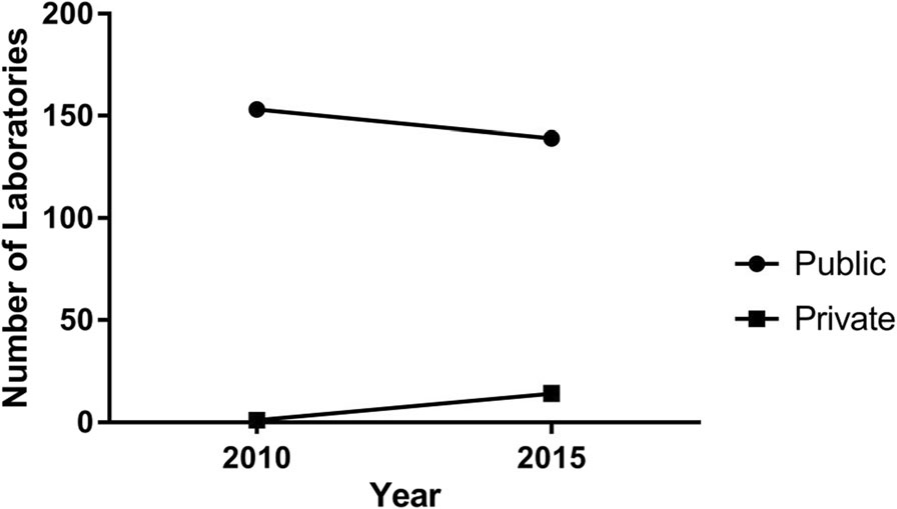
Role of Private providers. Data provided for 2015 show an increased role of private providers (bottom line), now equivalent to 13% of the total pathology budget

There was a minimal role for the private sector in the NHS pathology market in 2010. At the time, only one hospital used a private provider for its pathology services and all other laboratories were public. However, the figures are different when comparing the data for 2015. A number of hospitals (14 = 9% of the total, half of which are in London) are now partially managed by a private provider. A joint venture (between private and NHS) is present in 10 Trusts, whilst the other 4 Trusts have completely outsourced their pathology services. The total value for these pathology services is £237,223,655, equivalent to 13% of the total national pathology budget (Fig. 3).

### Impact on pathology staff

**Fig. 4:**
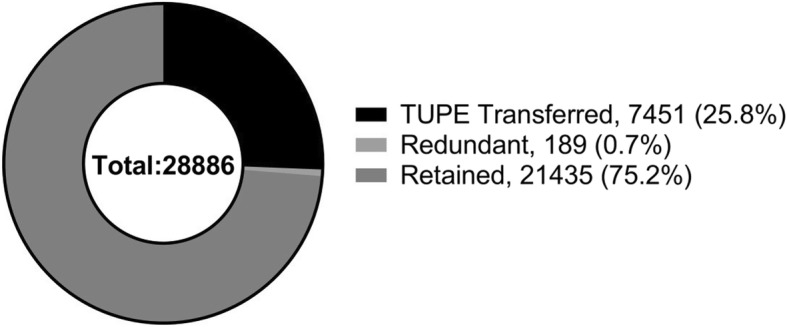
Impact of consolidation of laboratory services on pathology staff. In the last 5 years, there has been a minimal number of redundancies (0.7%) but 25.8% of staff have been TUPE transferred to a new organization

The total number of the pathology workforce in England is estimated to be 28,886 people (including all original 153 organizations), with various levels of experience from laboratory assistants to medical consultants. As a consequence of consolidation, 41 organizations (27% of the total) had to make some redundancies as part of their service reconfiguration. Nationwide, there have been 189 redundancies in the last 5 years, equivalent to 0.7% of the total workforce. Additionally, 46 Trusts (31%) have TUPE (transfer of undertakings) transferred their pathology staff, and this has affected 7451 people (26% of the total pathology workforce) (Fig. 4). The TUPE transfer can have an impact on pension contribution, in particular if personnel are transferred to a private provider. Only NHS staff is allowed to be part of the generous NHS pension scheme and transfer to another provider may cause a reduction in the final benefits.

## Discussion

The *hub-and-spoke* model has been implemented as part of the consolidation process of pathology services in England. Our analysis seems to confirm some of the annual national savings forecasted by the Carter’s reviews, but only when taking into consideration inflation and increased activity. Further analysis with stronger research design is required to confirm the role of consolidating laboratories in achieving financial savings. This acquires even more importance in view of the new push for pathology consolidation in England: new pathology networks have been identified by NHS improvement and these are expected to save the NHS at least £200 million pounds by 2020–2021 [23]. Previous Carter’s reviews [18, 19] also made a strong case supporting the consolidation of pathology services to improve quality and patient safety. However, the impact on quality and patient safety still need to be demonstrated. The lack of pathology tariffs is an anomaly in the NHS funding streams. Examples from the US and Europe have demonstrated how the reduction of the reimbursement rate (or tariff) was one of the major drivers in shaping the consolidation and this could explain the slow pace of change that has affected pathology services in England. Also, the introduction of tariffs could potentially increase quality. A fixed national tariff can be eventually reduced to drive efficiency savings but, at the same time, the various providers would only compete on quality of service and clinical advice, rather than costs.

The consolidation of pathology services has been paralleled by an increase in private sector involvement, now reaching 13% of the total pathology budget. This is mostly due to the lack of funds of many NHS hospitals and the initial capital investment necessary for the consolidation process. The interest of private sector in providing pathology services should not come as a surprise. The total pathology budget is worth more than 2 billion pounds and there is a wide range of technology and diagnostic companies that would like a share of it. However, this involvement is still limited when compared to other countries, in particular USA, Australia and Germany. Further analysis is needed to assess the role of private providers in the English NHS, in terms of advantages and disadvantages.

It has already been mentioned that savings in pathology generally result from the economy of scale and higher purchase discounts. Our analysis shows that the impact of consolidation on pathology staff has been minimal in terms of redundancies, with only 0.7% being affected. However, a fourth of the total workforce has been transferred to another organization, with potential changes in contract conditions and pension contributions. Data from telecommunication industries [24] have shown that mergers can decrease the workforce morale, affect the overall productivity (with low work performance and increased errors) and the relationships among co-workers. Unfortunately, there have been no studies on the impact of laboratory consolidation on the retention of specialized staff and the quality of pathology services. This was outside the remit of this project but it is a potential issue that would be worth further investigation.

Finally, it is important to consider the limitations of our study. The consolidation of pathology services in England is a dynamic and complex event, involving hundreds of laboratories and an evolving process. A direct comparison *like-for-like* may not always be possible due to recent mergers and closure of some laboratories. We have performed a simple descriptive comparison of pathology budgets in two different periods (2010 versus 2015) to reflect the majority of consolidations and, inevitably, some assumptions were made (inflation and increased activity). Such calculation did not consider the national cost improvement targets and it does not actually demonstrate the direct link between consolidation of pathology services and savings. This is to provide the reader with the actual data with minimal manipulation and the overall picture of the national pathology budget in relation to the forecasted savings proposed in the original Carter’s review. With this in mind, we would welcome debate and further analysis to independently evaluate the impact of pathology consolidation on both savings and quality.

## Conclusion

Consolidated units have on average achieved larger cost savings than non-consolidated units’, but there is a small minority of providers that have actually experienced a deficit, even when taking into consideration inflation and increased activity. In addition, the consolidation of services has opened the pathology market to the private sector, with an increased number of private laboratories operating in the last 5 years. Our analysis is unable to directly prove that the greater cost savings are due to the consolidation process but savings were achieved with negligible redundancies. The long-term impact on the pathology workforce and the quality of pathology services is worth further investigation.
